# Data Analysis and Tissue Type Assignment for Glioblastoma Multiforme

**DOI:** 10.1155/2014/762126

**Published:** 2014-03-03

**Authors:** Yuqian Li, Yiming Pi, Xin Liu, Yuhan Liu, Sofie Van Cauter

**Affiliations:** ^1^School of Electronic Engineering, University of Electronic Science and Technology of China, Chengdu 611731, China; ^2^Department of Radiology and Department of Imaging and Pathology, University Hospitals of Leuven, 3001 Leuven, Belgium

## Abstract

Glioblastoma multiforme (GBM) is characterized by high infiltration. The interpretation of MRSI data, especially for GBMs, is still challenging. Unsupervised methods based on NMF by Li et al. (2013, *NMR in Biomedicine*) and Li et al. (2013, *IEEE Transactions on Biomedical Engineering*) have been proposed for glioma recognition, but the tissue types is still not well interpreted. As an extension of the previous work, a tissue type assignment method is proposed for GBMs based on the analysis of MRSI data and tissue distribution information. The tissue type assignment method uses the values from the distribution maps of all three tissue types to interpret all the information in one new map and color encodes each voxel to indicate the tissue type. Experiments carried out on *in vivo* MRSI data show the feasibility of the proposed method. This method provides an efficient way for GBM tissue type assignment and helps to display information of MRSI data in a way that is easy to interpret.

## 1. Introduction

Glioblastoma multiforme (GBM), which typically consists of three tissue types (i.e., normal, tumor, and necrosis), is a type of extensively heterogeneous tumors. Accurate diagnosis of GBMs is of great importance for guiding therapy and planning operations. Being different from other brain tumors which present similar spectral patterns, GBMs are characterized by high infiltration [1, 2]. Such characteristic brings huge difficulty in tumor typing and diagnosis.

Magnetic resonance spectroscopy imaging (MRSI) [3] is a very useful noninvasive tool for brain tumor diagnosis, especially for highly heterogeneous tumors like GBMs. Unlike magnetic resonance imaging (MRI) which only shows the brain structure, MRSI combines MRI and magnetic resonance spectroscopy (MRS) [4] to provide the localized biochemical information. By investigating the spectra from multivoxels, the clinicians could have a better insight into the pathological change of brain tissues.

However, the interpretation of MRSI data is still challenging which hinders its application in tumor diagnosis. Efforts for exploiting MRSI data have been made using both supervised and unsupervised methods. Nosologic imaging is created using linear discriminant analysis [5, 6], canonical correlation analysis (CCA) [7, 8], Bayesian frameworks [9, 10], and nonnegative matrix factorization (NMF) [11]. NMF [11] is an alternative blind source separation technique with only nonnegative constraint. It has shown great potentials in brain tissue differentiation [2, 12–14]. In our previous work, we proposed an unsupervised method, namely, hierarchical nonnegative matrix factorization (hNMF), to interpret the MRSI data for GBMs without prior knowledge and provided an easy way to interpret MRSI data of GBMs for each tissue type [15].

Unlike the supervised classification methods, which labels each voxel based on large training sets [5–10], tissue typing for NMF tissue differentiation is not usually considered [12, 13, 16]. Recently, a tissue typing method was carried out by simply exploring which tissue contributes most to the voxel [14]. Such an approach ignored the voxels with intensively mixed tissues, that is, the different tissues contributing fairly equal. We tried to integrate the distribution information of each pure tissue in one image by encoding each of them as a color channel [16]. The obtained images, known as nosologic images, showed the spatial distribution of all tissue types. However, the tissue distribution is only shown in shading colors and the tissue type of each voxel is not indicated clearly.

In this paper, we improved upon [15] by proposing an approach for GBM tissue type recognition. The previous work is extended by analyzing both the pure and mixed data labeled by an expert. The spectral data labeled as each tissue type is analyzed and the relationship of different tissue types is studied. Then, we proposed criteria to assign each voxel to a certain tissue type (i.e., pure tissue normal, tumor, necrosis, mixed tissues normal/tumor, or tumor/necrosis, hereafter noted as “C”, “T”, “N”, “C/T”, and “T/N,” resp.) using the tissue distribution maps. *In vivo* experiments are performed using short-TE ^1^H MRSI data from GBM patients. We then evaluate its performance using the expert labeling.

## 2. Materials

### 2.1. Data Acquisition Protocol

The MRSI protocol had the same imaging parameters as in our previous work [15, 16]. All the MRSI data were acquired at the University Hospital of Leuven (UZ Leuven, Belgium) on a 3 T MR scanner (Achieva, Philips, Best, The Netherlands). A body coil for transmission and eight-channel head coil for signal reception were used. The MRSI protocol had the following imaging parameters: point-resolved spectroscopy (PRESS) [17] that was used as the volume selection technique; TR/TE = 2000/35 ms; field of view, 16 cm × 16 cm; volume of interest, 8 cm × 8 cm (maximum size); slice thickness, 1 cm; acquisition voxel size, 1 cm × 1 cm; reconstruction voxel size, 0.5 cm × 0.5 cm; receiver bandwidth, 2000 Hz; samples, 2048; number of signal averages, 1; water suppression method, MOIST; shimming, pencil beam shimming; first- and second-order parallel imaging with SENSE factor: left-right, 2; anterior-posterior, 1.8; 10 circular 30 mm outer-volume saturation bands in order to avoid lipid contamination from the skull. Standard anatomical MR images were also acquired.

### 2.2. Patients and Data

MRSI data sets from 6 GBM patients (typically present three tissue patterns, i.e., normal, tumor, and necrosis) were selected for this study. The MRSI data was acquired prior to any treatment from 6 patients with brain tumors that were subsequently diagnosed as GBM based on histological examination and followed the rules of the World Health Organization (WHO) classification for tumor grading [18]. The institutional review board approved the study. Written informed consent was obtained from all patients before their participation in the study. Data preprocessing was done as in our previous papers [15, 16] using the in-house software SPID [19].

### 2.3. Expert Labeling

MR spectra were judged by a spectroscopist (a radiologist with five years of experience). The expert spectroscopist was presented with the real spectra in a range from 4.3 to 0 ppm.

Firstly, spectral quality assessment was performed as recommended by Kreis [20]. Spectra were judged acceptable if the following criteria were met: FWHM of metabolites < 0.07–0.1 ppm, no unexplained features in the residuals, no doubled peaks or evidence for movement artifacts, symmetric lineshape, and no outer-volume ghosts or other artifacts present.

Afterwards, the spectra with acceptable quality were assigned to different tissue classes: normal appearing brain parenchyma, tumoral tissue, or necrosis, based on the spectra and the corresponding T1-weighted image after contrast administration.

## 3. Method

### 3.1. Spectra Investigation for GBMs Using Biomarkers

N-acetylaspartate (NAA), choline (Cho), and lipids are known to be the three most important biomarkers for investigating brain tumorigenesis. The concentration of these metabolites changes under disease condition. In the context of GBM spectroscopy, necrosis mostly contains lipids. NAA concentration is higher than Cho in normal tissue and gliomas are characterized by decreased NAA and increased Cho and lipids. But these biomarkers are not enough for MRSI spectra differentiation. In a specific frequency region of a spectrum, the peak height of the metabolite can be measured. Here, we use the NAA-to-Cho index (NCI) and NAA-to-Lips index (NLI), which measure the ratios of the peak heights of these components, to investigate the spectra for all GBM patients. We select all voxels containing pure tissues and mixed tissues to observe if the biomarkers are capable of clustering the same tissues. Each point represents a spectrum from a single voxel. Its coordinate values (*x*, *y*) correspond to the NCI value and NLI value, respectively. The points are colored using expert labeling to indicate their tissue types, blue for “C,” cyan for “C/T,” green for “T,” yellow for “T/N,” and red for “N.”

### 3.2. Spectra Variation Investigation Using Expert Labeling

In this section, we investigate the relationship of spectral variation and expert labeling, including two aspects: (1) the variation of spectra labeled as the same tissue type and (2) the variation of spectral difference between two tissue types.

The expert labeled spectrum in each voxel as a certain tissue type “C,” “T,” “N,” “C/T,” and “T/N.” However, because of the voxel size of MRSI data and the infiltration property of GBMs, there is no clear boundary between different tissue types, especially in the area of tumor proliferation. Therefore, the spectra labeled as the same tissue type could have different profiles. In order to investigate the spectra variation, we plot all spectra of each pure tissue type and their mean spectra.

The correlation coefficients between normal and tumor spectra *R* ^CT^ and the correlation coefficients between tumor and necrotic spectra *R* ^TN^ can evaluate the spectral difference of different tissue types. For each spectrum labeled as a pure tissue type, we calculate the correlation coefficient of this spectrum and a spectrum labeled as another pure tissue type. With box plots, the variation of spectral difference between two different tissue types could be observed easily. Combined data of all patients and also that of individual patients are both analyzed to investigate the spectral difference between different tissue types.

### 3.3. Tissue Differentiation with hNMF

Spectra from a MRSI grid can be approximated as a linear combination of *r* constituent spectra. We define a data matrix *X* containing all spectra from the voxels of interest (VOI). Each column of matrix *X* represents a spectrum from one voxel. With conventional NMF, *X* can be factorized into a new nonnegative matrix *W* (each row represents a constituent spectrum of normal tissue or necrosis) and a new nonnegative matrix *H*,

(1) Xm×n≈Wm×rHr×nsubject  to  W,H≥0.

The reshape of each row of *H*, hereafter called “*h*-map,” that is, the “tissue distribution maps” we mentioned before, gives the spatial distribution of the corresponding spectrum.

For the low grade gliomas, conventional NMF is able to differentiate normal and abnormal (i.e., tumor) tissues. While there are more than two tissue types (e.g., GBMs), the conventional NMF sometimes fails to recover the biomeaningful constituent spectra robustly. Therefore, a hierarchical approach based on NMF (i.e., hNMF) was proposed to recover the spectra of MRSI data for GBMs which contains 3 constituent spectra [15]. HNMF firstly differentiates the data matrix into normal and abnormal. Then, with an optimized threshold, the abnormal part is further differentiated into tumor and necrosis. As a result, the three constituent spectra of normal, tumor, and necrosis are recovered and their *h*-maps for different tissue types are obtained simultaneously. Note that, in each voxel, there are 3 values from the 3 *h*-maps *h*_*i*_^C^, *h*_*i*_^T^, and *h*_*i*_^N^ for each tissue type.

### 3.4. *h*-Map Investigation Using Expert Labeling

As introduced in the previous section, the *h*-maps, which are normalized between 0 and 1, can be obtained from the result of hNMF. Then, the *h*-map of each tissue type represents the tissue distribution using a number for a voxel. However, during expert labeling, each voxel is arbitrarily labeled as a certain tissue type instead of a number. It is obvious that the *h*-values (hereafter, the value from a single voxel in an *h*-map referred to as “*h*-value”) from the voxels, which are labeled as the same tissue type, could be different. In this section, we will exploit the *h*-values of each tissue type. *H*-values of all patients and each patient are also exploited to reveal the extent of individual difference.

### 3.5. Tissue Type Assignment

Based on the data analysis of *h*-maps, each voxel could be assigned to a certain tissue type. In each voxel, there are 3 *h*-values from 3 *h*-maps for “C,” “T,” and “N,” respectively. Obviously, the *h*-values of “C” (i.e., *h*_*i*_^C^) should be bigger than the *h*-values of “T” (i.e., *h*_*i*_^T^) and the *h*-values of “N” (i.e., *h*_*i*_^N^) for normal voxels. Analogically, for voxels of tumor and necrosis tissue, *h*_*i*_^T^ and *h*_*i*_^N^ should overwhelm *h*-values of other tissue types, respectively.

However, there are mixed tissues where *h*-values of each tissue type vary significantly and thus the above criteria cannot be simply used to decide the tissue type. To properly separate the different tissues from the mixed tissues, a parameter *ρ* should be added; that is, *h*_*i*_^C^ should be bigger than *h*_*i*_^T^ + *ρ* ^CT^ for the voxel to be assigned to be “C.” Similarly, *h*_*i*_^N^ should be bigger than *h*_*i*_^T^ + *ρ* ^TN^ for the voxel to be assigned to be “N.” Therefore, we make the following criteria for tissue type assignment.

*The Rules for Tissue Type Assignment*

 While *h*_*i*_^C^ > *h*_*i*_^T^, *h*_*i*_^C^ > *h*_*i*_^N^, and *h*_*i*_^C^ > *h*_*i*_^T^ + *ρ* ^CT^, assign the voxel to be “C”;
 While *h*_*i*_^N^ > *h*_*i*_^T^, *h*_*i*_^N^ > *h*_*i*_^C^, and *h*_*i*_^N^ > *h*_*i*_^T^ + *ρ* ^TN^, assign the voxel to be “N”;
 While *h*_*i*_^T^ > *h*_*i*_^C^ + *ρ* ^CT^ and *h*_*i*_^T^ > *h*_*i*_^N^ + *ρ* ^TN^, assign the voxel to be “T”;
 Else if *h*_*i*_^N^ < *h*_*i*_^C^ and *h*_*i*_^N^ < *h*_*i*_^T^, assign the voxel to be “C/T”;
 Else if *h*_*i*_^C^ < *h*_*i*_^T^ and *h*_*i*_^C^ < *h*_*i*_^N^, assign the voxel to be “T/N.”

According to the above criteria, we can have all the voxels assigned to a certain type, including the ones originally labeled as “B” by expert.

### 3.6. Validation

The efficacy of the proposed tissue type assignment approach is validated using expert labeling information. The computed tissue type of each voxel is compared with the tissue type labeled by expert. We use the correct rate, false alarm rate, and the omission rate to evaluate the performance of the proposed approach.

Correct rate describes correct assignment among all the assignment,

(2) $$\begin{array}{c} \text{Correct}\;\text{rate}=\frac{N_{\text{correct}}}{N_{\text{assigned}}}, \end{array}$$

where *N*_assigned_ represents the number of voxels which are assigned to a certain tissue type using the proposed method. And *N*_correct_ represents the number of voxels assigned to a certain tissue type that our assignment is the same as that of an expert.

False alarm rate describes the wrong assignments which should not be counted,

(3) $$\begin{array}{c} \text{False}\;\text{alarm}\;\text{rate}=\frac{N_{\text{error}}}{N_{\text{assigned}}}, \end{array}$$

where *N*_error_ represents the number of voxels which are assigned to be a certain tissue type using the proposed approach but not labeled by an expert as the same tissue type.

Omission rate describes the wrong assignment which is missed,

(4) $$\begin{array}{c} \text{Omission}\;\text{rate}=\frac{N_{\text{omission}}}{N_{\text{assigned}}}, \end{array}$$

where *N*_omission_ represents the number of voxels which are labeled by an expert to be a certain tissue type but not assigned as the same tissue type using the proposed approach.

## 4. Results and Discussion

### 4.1. Spectra Investigation for GBMs Using Biomarkers

**Figure 1:**
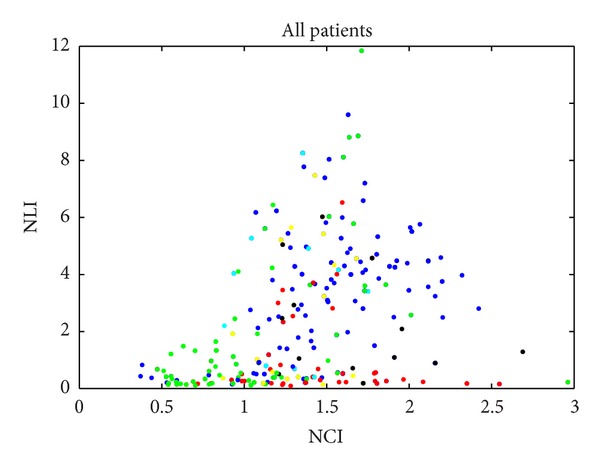
Investigation of *in vivo* ^1^H MRSI data from GBM patients. *x*-axis is the NAA-to-Cho index (NCI) and *y*-axis is the NAA-to-Lips index (NLI). Blue, green, and red points indicate normal, tumor, and necrotic tissue, respectively. Mixed colors represent mixed tissue. Blue for “C,” cyan for “C/T,” green for “T,” yellow for “T/N,” and red for “N.”

We investigated all the 6 data sets which were pathologically confirmed to be GBM by clinicians.  Figure 1 shows the overall tissue types of all the GBM data sets. Each point represents a spectrum from one voxel among all the data sets. The points are colored using expert labeling, same color for same tissue type. Due to the variation of the spectra, the distribution map shows serious overlap between tissue types. Though there are two vaguely centralized clusters for normal (higher NLI and NCI) and necrosis (very low NLI and lower NCI), there are no clear dividing lines between tissue types. Tumor cannot be separated from normal and necrosis. Mixed tissues cannot be differentiated from other tissue types, either.

### 4.2. Spectra Variation Investigation Using Expert Labeling

**Figure 2:**
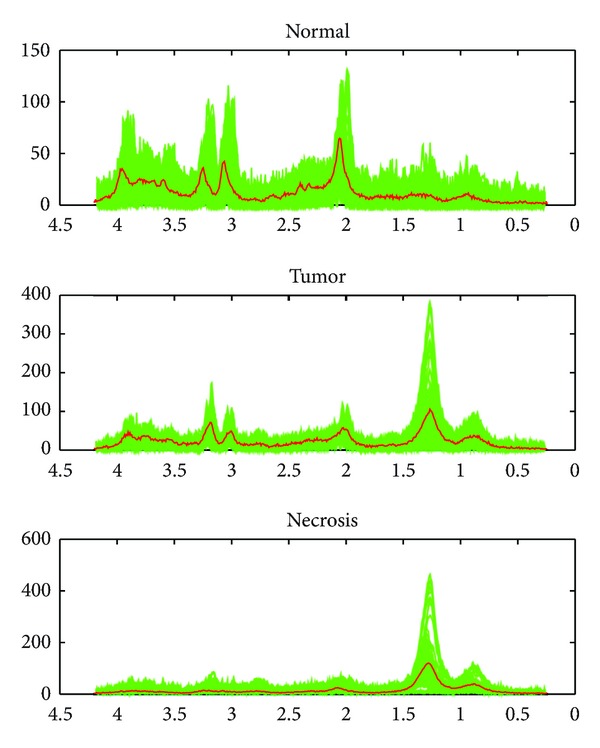
Spectral variation of pure tissues. Each green spectrum is from a voxel. The red bold line represents the mean spectrum.

The spectral variation of pure tissues labeled by an expert is investigated by plotting all spectra of the same tissue from all GBM patients in one figure. As shown in Figure 2, the green spectra are from all the voxels labeled as normal, tumor, and necrosis by an expert. Serious spectral variations for the same tissue type can be observed. It demonstrates that spectra for the same tissue type are possibly not identical. The red bold line plots the mean spectrum for normal, tumor, and necrosis, respectively. We can observe that most of the green spectra have great difference with the mean spectra.

**Figure 3:**
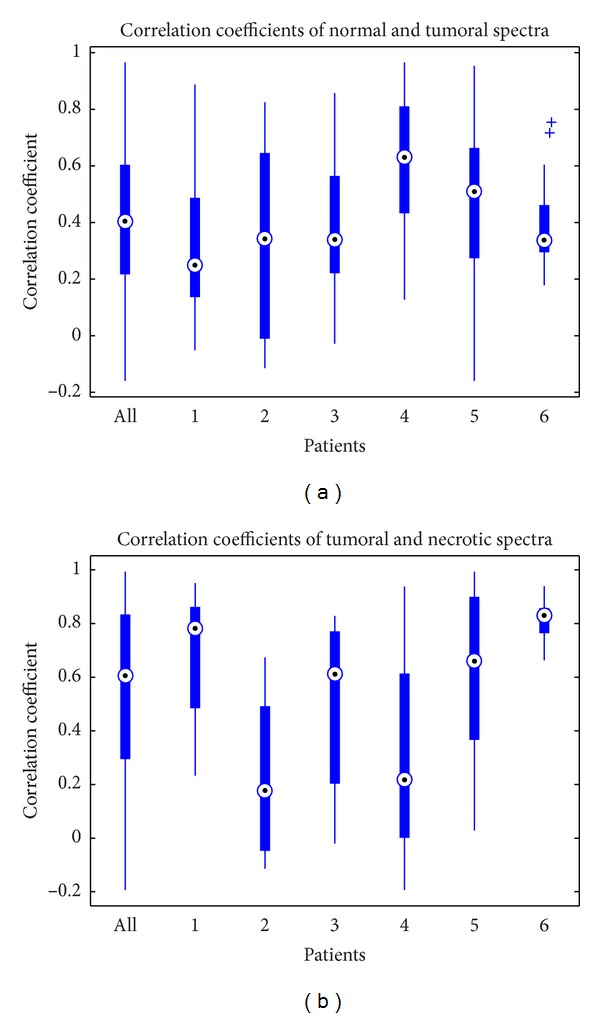
Correlation coefficients of different spectra.

The spectral relationships of different tissue types are investigated using correlation coefficients. The correlation coefficients of each spectrum labeled as “C” by expert and the spectrum labeled as “T” by expert, noted as *R* ^CT^, are calculated to investigate the difference of normal spectra and tumor spectra and its variation.  Figure 3(a) shows the *R* ^CT^ for all the GBM patients and each individual patient. As shown, most of the correlation coefficients *R* ^CT^ are between 0.3 and 0.7. However, some values are extremely small or big because of the variation of tumor spectra. Differences between patients are not significant except for two patients, that is, patients 4 and 6. It demonstrates that the serious variation among patients is not common but possible. The lower quartile of *R* ^CT^, *Q*1^CT^ = 0.2167, could be used to describe the relationship between normal and tumor.

Similarly, the correlation coefficients of each spectrum labeled as “T” by expert and the spectrum labeled as “N” by expert, noted as *R* ^TN^, are calculated to investigate the difference of tumor spectra and necrotic spectra and its variation, as shown in Figure 3(b). Compared to *R* ^CT^, the variation of *R* ^TN^ is more serious. However, the *R* ^TN^ values of all patients inside the box are between 0.3 and 0.8. The lower quartile of *R* ^TN^, *Q*1^TN^ = 0.2950, could be used to describe the relationship between tumor and necrosis.

### 4.3. *h*-Maps Variation for Different Tissue Types

**Figure 4:**
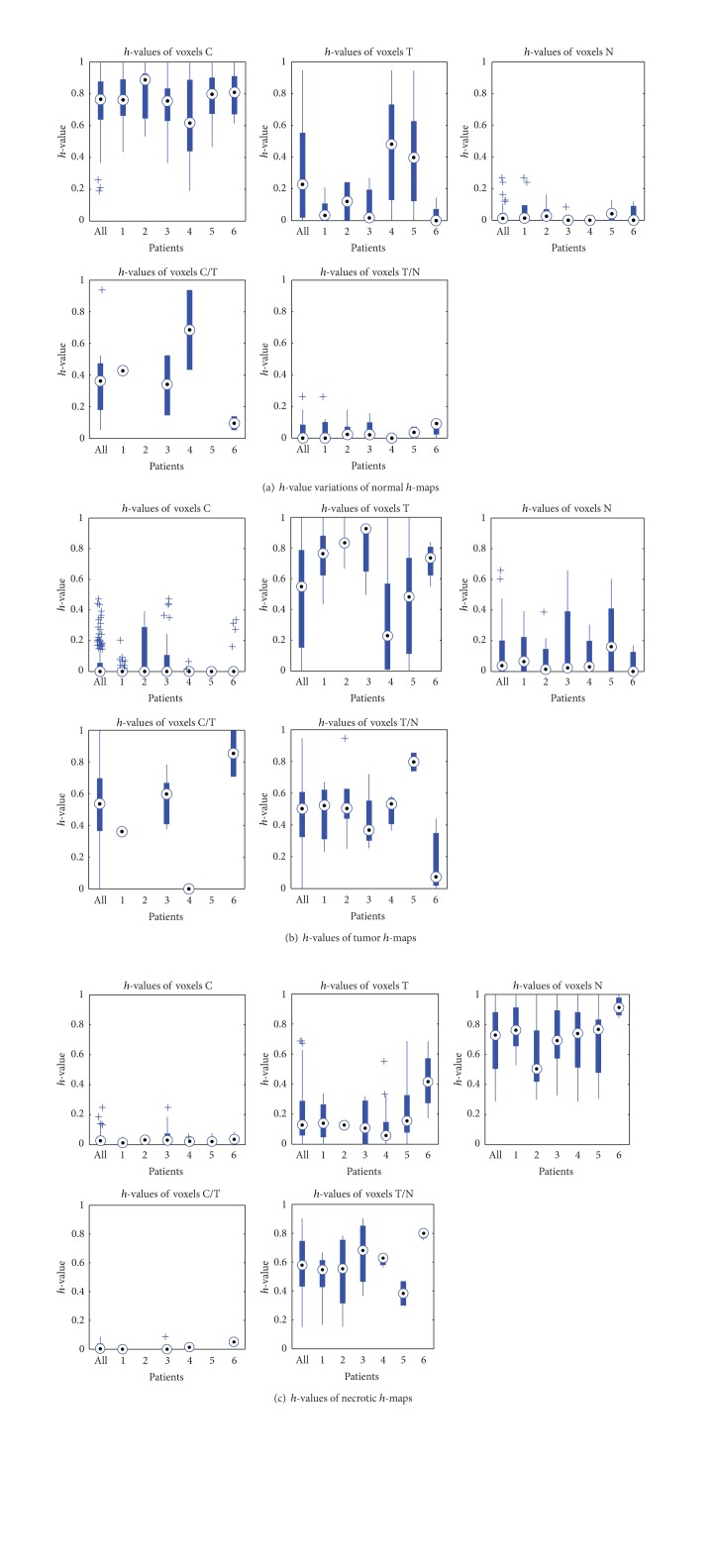
Variations of *h*-values of intra- and interpatients.

The values in *h*-maps for each labeled specific tissue type are analyzed.  Figure 4 gives the *h*-values from the 6 GBM patients. For the 6 data sets, there are 6 normal *h*-maps, 6 tumor *h*-maps, and 6 necrosis *h*-maps. For *h*-maps of each tissue type, we analyzed the data distribution of tissue types for all patients.

Figure 4 illustrates the *h*-values of normal *h*-map. Each plot contains a box for all patients and 6 boxes for 6 GBM patients.  Figure 4(a) depicts the *h*-values taken from *h*-maps of normal tissue. The values from voxels labeled as “C,” “T,” “N,” “C/T,” and “T /N” are depicted. As shown, the values for “C” are mostly between 0.6 and 1. The values for “N” and “T/N” are all quite small. It implies that good separation of normal and necrosis is possible using *h*-maps. For the other two types “T” and “T/N”, the values vary greatly.  Figure 4(b) depicts the *h*-values taken from tumor *h*-maps. As shown, values for all tissue types vary seriously, even for tumor.  Figure 4(c) depicts the *h*-values taken from necrosis *h*-maps. The values for “N” are mostly between 0.5 and 0.9. The values for “C” and “C/T” are quite small. It also implies that good separation of normal and necrosis is possible using *h*-maps. The values for “T” and “T/N” vary greatly. In general, the *h*-values taken from tumor *h*-maps vary more seriously than the *h*-values taken from *h*-maps of normal and necrosis, and the values for “T,” “C/T,” and “T/N” taken from all three *h*-maps vary significantly. It implies that the separation of tumor and mixed tissue is more difficult than normal and necrosis.

### 4.4. Tissue Type Assignment for GBMs

**Figure 5:**
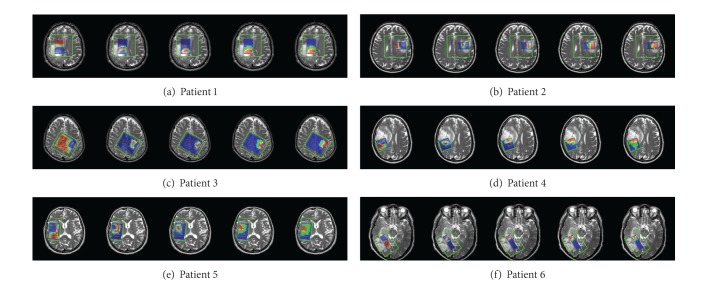
*h*-maps and tissue type assignment results compared with expert labeling. All the results are overlaid on the T2-weighted MRI. For each patient, the first 3 images are the *h*-maps for normal, tumor, and necrosis. The fourth image is the assigned tissue types. The last image is the expert labeling.

The proposed tissue type assignment method described in Section 3.5 is applied to the *h*-maps of 6 GBM data sets. *Q*1^CT^ = 0.2167 and *Q*1^TN^ = 0.2950 are used as *ρ* ^CT^ and *ρ* ^TN^, respectively. The results are compared with expert labeling information. For both the results and the expert labeling, the distribution map is color-coded blue for “C,” cyan for “C/T,” green for “T,” yellow for “T/N,” red for “N,” and black for “B” which is spectra of low quality of which the tissue type cannot be decided by expert. As shown in Figure 5, the assigned tissue types are approximately in accordance with the expert labeling. The regions of normal and necrosis are more accurate than the regions of tumor and mixed tissues like “C/T” and “T/N.” This is mainly because the high infiltration character of gliomas brings higher variation to the spectral profiles of tumor and mixed tissues. The black voxels labeled as “B” by expert can be estimated using the proposed method. After analyzing localization of these voxels and their surrounding voxels, the assignment of these voxels was confirmed to be correct.

### 4.5. Validation

For each patient, the correct rate, false alarm rate, and the omission rate are calculated for each pure tissue and mixed tissue types by comparing results with expert labeling. The “N/A” in Table 1 represents the situations which do not exist.

As we observe, the assignments of pure tissue “C” and “N” are almost always more accurate than “T.” The correct rate of “C” and “N” can be as good as above 0.9 or even 1. The correct rate of mixed tissues (i.e., C/T and T/N) is lower than pure tissues.

The omission rates of the pure tissue “C” and “N” for all patients are lower than 0.5, mostly lower than 0.4. But for “T” and mixed tissue “C/T,” “T/N,” the omission rate is higher.

For all results, the pure tissues “C” and “N” perform better than “T” and “T” performs better than the mixed tissues “C/T” and “T/N.” Inaccurate assignment of a tissue type influences the assignment of the tissues near it. In other words, the correct rate or error rate of “C,” “T,” and “N” will be affected by the inaccurate assignment of “C/T” and “T/N.”

## 5. Discussions

This study continued with our tissue typing work using hNMF [15]. We explored the possibility of only using several most representative biomarkers for tissue differentiation. The results showed that the different tissue types cannot be well separated, especially for tumor and mixed tissues. Therefore, a new approach for tissue type assignment using hNMF is developed.

Then we evaluated the relationship between spectra of different tissue types. The spectra labeled as a certain tissue type by expert are compared to the spectra labeled as another tissue type. The variation of the different correlation coefficients for both intra- and interpatient indicates the difference of spectra which are labeled as the same tissue type. This implies that the spectra are not identical even if they are labeled as the same tissue type, especially for tumoral spectra. This could be due to the fact that glioblastoma are known to be very heterogeneous lesions. Invasion, regions of increased cellularity, necrosis on a microscopic and a macroscopic scale, hemorrhage, and microvascular proliferation are hallmarks of the most malignant of gliomas. This heterogeneity is reflected in the variation of the spectra, related to tumoral tissue. A voxel in the chemical shift imaging (CSI) protocol used in this study is approximately 0.25 cm^3^. Thus, thousands of metabolites will contribute to the measured signal. The spectra in MRS are only indirect indicators of metabolism. For example, regions of tumoral tissue are characterized by high cellularity and are perceived as spectra with strongly elevated choline and decreased NAA. Regions with tumoral tissue with necrosis on a microscopic scale will be perceived with moderately elevated lipids and lower values of choline and NAA. Tumoral regions with moderately elevated cellularity will be perceived as regions with only moderately elevated Cho and moderately lowered NAA. In the end, these spectra represent all tumoral tissue, as designated by the histopathologist as well as by the expert labeling in MR spectroscopy [21–25]. Therefore, spectral variation within the same tissue type, which is introduced by the nature of tumor proliferation and the volume of CSI voxels, could happen and influence the performance of tissue typing method.

As demonstrated, the lower quartiles of correlation coefficients *Q*1^CT^ and *Q*1^TN^ could imply the “least spectral similarity” between different tissue types. Additionally, the scale of correlation coefficients *R* ^CT^ and *R* ^TN^ is in the same scale of *h*-values. Therefore, in the tissue typing assignment experiment, where *Q*1^CT^ = 0.2167 and *Q*1^TN^ = 0.2950, which were calculated using 6 GBM patients, were used as the parameters *ρ* ^CT^ and *ρ* ^TN^, respectively. Though the patients were few, the value of *Q*1^CT^ and *Q*1^TN^ will not change significantly since the voxel number for calculating them is large enough to be stable. Another important point we must stress is that, as long as we have decided the value for parameters *ρ* ^CT^ and *ρ* ^TN^, we do not need to calculate *Q*1^CT^ and *Q*1^TN^ every time there is a new patient. The tissue assignment method is still automatic since the values used as *ρ* ^CT^ and *ρ* ^TN^ will be fixed numbers. Here, we just proposed a potential way to decide *ρ* ^CT^ and *ρ* ^TN^.

As the tissue type assignment is based on the *h*-maps of hNMF, the results could be affected by both the *h*-maps and the typing criteria. As shown, the results for tumor and mixed tissues are worse than the results for normal and necrosis. On the one hand, there is the serious variation of tumor spectra. On the other hand, the spectral profile of C/T and T/N is highly correlated with the tumor spectra. These facts lower the typing results. However, the assignments of each tissue type shown in Section 4.4 have shown the efficacy of the proposed method.

## 6. Conclusions

In this paper, we investigate the spectra variation with expert's labeling. Tissue type assignment criteria are proposed to assign each voxel to 5 different tissue types, including 3 pure tissue types “C,” “T,” and ”N” and 2 mixed tissue types “C/T” and “T/N,” using the *h*-maps of normal, tumor, and necrosis obtained by hNMF. Experiments show the feasibility of the proposed method for tissue type assignment.

## Figures and Tables

**Table 1:** Result validation.

	C	T	N	C/T	T/N
Patient 1					
Detected number	38	12	13	5	9
Correct detected number	38	11	12	0	6
Number of voxels labeled by expert	38	19	12	0	8
Correct rate	1	0.9167	0.9231	0	0.6667
Error rate/false alarm rate	0	0.0833	0.0769	1	0.3333
Omission rate	0	0.4210	0	N/A	0.2500
Patient 2					
Detected number	14	4	13	2	7
Correct detected number	12	3	13	1	5
Number of voxels labeled by expert	13	3	14	0	7
Correct rate	0.8571	0.7500	0.9231	0.5000	0.7142
Error rate/false alarm rate	0.1429	0.2500	0.0769	0.5000	0.2857
Omission rate	0.0769	0	0.1429	N/A	0.2857
Patient 3					
Detected number	108	13	9	9	4
Correct detected number	108	5	7	3	2
Number of voxels labeled by expert	115	5	11	6	6
Correct rate	1	0.3846	0.7778	0.3333	0.5000
Error rate/false alarm rate	0	0.6153	0.2222	0.6667	0.5000
Omission rate	0.0608	0	0.3636	0.5000	0.6667
Patient 4					
Detected number	27	4	9	7	9
Correct detected number	14	4	9	1	4
Number of voxels labeled by expert	16	21	11	4	4
Correct rate	0.5185	1	1	0.1429	0.4444
Error rate/false alarm rate	0.4815	0	0	0.8571	0.5556
Omission rate	0.1250	0.8095	0.1818	0.7500	0
Patient 5					
Detected number	38	19	10	4	6
Correct detected number	20	17	8	0	0
Number of voxels labeled by expert	20	44	11	0	2
Correct rate	0.5263	0.8947	0.8000	0	0
Error rate/false alarm rate	0.4737	0.1053	0.2000	1	1
Omission rate	0	0.6136	0.2727	N/A	1
Patient 6					
Detected number	21	5	6	0	1
Correct detected number	21	3	3	0	0
Number of voxels labeled by expert	21	4	3	2	3
Correct rate	1	0.6000	0.5000	N/A	0
Error rate/false alarm rate	0	0.4000	0.5000	N/A	1
Omission rate	0	0.2500	0	1	1
